# Sarcopenia: Diagnosis and Management, State of the Art and Contribution of Ultrasound

**DOI:** 10.3390/jcm10235552

**Published:** 2021-11-26

**Authors:** Silvia Giovannini, Fabrizio Brau, Raffaele Forino, Andrea Berti, Federica D’Ignazio, Claudia Loreti, Andrea Bellieni, Emanuela D’Angelo, Francesca Di Caro, Lorenzo Biscotti, Daniele Coraci, Augusto Fusco, Luca Padua, Roberto Bernabei

**Affiliations:** 1 Department of Geriatrics and Orthopaedics, Università Cattolica del Sacro Cuore, 00168 Rome, Italy; febrisb@gmail.com (F.B.); raffaeleforino@hotmail.it (R.F.); andbe.med@gmail.com (A.B.); federica.dignazio20@gmail.com (F.D.); emanuela.dangelo@policlinicogemelli.it (E.D.); francesca.dicaro@policlinicogemelli.it (F.D.C.); luca.padua@unicatt.it (L.P.); roberto.bernabei@unicatt.it (R.B.); 2 UOS Riabilitazione Post-Acuzie, Fondazione Policlinico Universitario A. Gemelli IRCCS, 00168 Rome, Italy; andrea.bellieni@policlinicogemelli.it; 3 Department of Aging, Neurological, Orthopaedic and Head-Neck Sciences, Fondazione Policlinico Universitario A. Gemelli IRCCS, 00168 Rome, Italy; claudia.loreti@policlinicogemelli.it (C.L.); lorenzo.biscotti@unicatt.it (L.B.); 4 Presiding Officer of Geriatric Care Promotion and Development Centre (C.E.P.S.A.G.), Università Cattolica del Sacro Cuore, 00168 Rome, Italy; 5 Department of Neuroscience, Section of Rehabilitation, University of Padova, 35122 Padua, Italy; danielecoraci@aol.com; 6 UOC Neuroriabilitazione ad Alta Intensità, Fondazione Policlinico Universitario A. Gemelli IRCCS, 00168 Rome, Italy; augusto.fusco@policlinicogemelli.it

**Keywords:** ultrasound, sonography, nerve, muscle, technology, diagnostic tool, neuropathies, myopathies, personalized medicine

## Abstract

Age-related muscle loss is a phenomenon that has been extensively studied in recent decades. Sarcopenia is a multisystem disease, which predisposes to muscle weakness and frailty. At around 50 years of age, an individual begins to lose muscle strength, although this becomes more evident after 70. Sarcopenia is a condition typically found in older adults but can also affect younger people. Sarcopenia is a preventable and treatable condition. In past years, methods and tools to recognize the condition early have been researched. For the development of therapeutic interventions, agreement on diagnosis is fundamental. In recent years, a possible role of ultrasonography in the diagnosis of sarcopenia has been evaluated, compared with the best-known techniques.

## 1. Introduction

Sarcopenia is a progressive, systemic musculoskeletal disorder associated with an increased risk of adverse events such as falls and fractures, mobility disorders, cardiac and respiratory disease, cognitive impairment, institutionalization, and death [1]. Physical disability and impaired ability to perform activities of daily living contribute to reducing both patient quality of life and functional independence, adding to the necessity of long-term care services for the patient [2,3]. Considering this evidence, it would seem clear that early diagnosis of sarcopenia and care optimization would also reduce the economic impact on the health care system and individual social-economic burdens [4]. Numerous studies and research groups have been working over the past ten years to provide a precise definition of sarcopenia. Five major research groups around the world have developed different definitions and diagnostic criteria: the European Working Group on Sarcopenia in Older People (EWGSOP), the Asia Working Group for Sarcopenia (AWGS), the International Working Group on Sarcopenia (IWGS), the Foundation for the National Institutes of Health (FNIH) Sarcopenia Project, and the Society for Sarcopenia Cachexia and Wasting Disorders (SCWD). As a result, in 2016, sarcopenia was added to the International Classification of Diseases with its ICD-10-MC diagnostic code [5,6]. In 2018, the European Working Group on Sarcopenia in Older People (EWGSOP2) published the revised “European Consensus on Sarcopenia” introducing a new working definition, based on low muscle strength as the primary parameter of sarcopenia, considering it a more reliable measure of muscle function and a better predictor of adverse outcomes. Behind sarcopenia, there are mechanisms that in some cases overlap with some cell aging processes. In particular, there are alterations in protein synthesis, proteolysis, neuromuscular integrity, and muscle fat content [7,8,9]. It would be considered that sarcopenia is common in significative fragile patients, often affected by multimorbidity, hospitalization, and consumption of several drugs [10,11].

Screening and diagnosis of sarcopenia are important to prevent adverse health outcomes. CT and MRI are the gold standards for noninvasively assessing muscle quantity/mass and identifying adipose tissue [12,13]. However, these examinations are expensive, expose patients to radiation, are not always available, and in some situations are difficult to perform and thus must be performed in the hospital. DXA is more easily accessible but less accurate [1].

In the last few years, new evidence has shown that ultrasound provides detailed imaging about morphology and size and of the surrounding structures [14]. Several studies reported ultrasound as an appropriate tool to evaluate muscle characteristics and morphology in sarcopenia [15,16,17].

Moreover, ultrasonography is low cost, simple to use, community or hospital-based, is easily repeatable, and is widely used.

The age-related biochemical, anatomical, and physiological changes in muscle tissue have been known for some time. These alterations occur at the molecular and cellular levels and are reflected in macroscopic changes evident on ultrasound. Alterations in muscle architecture reflect underlying sarcopenia, which causes muscle dysfunction. These changes have been recognized as good imaging biomarkers based on ultrasonography [18].

The quality and quantity of muscle tissue can be easily assessed with ultrasound by using some specific parameters.

In particular, the evaluation of pennate muscles is a potentially valuable tool. In clinical practice, it can highlight even minimal changes over a short period [19].

Ultrasonography is an accurate method even for use in the elderly, is easy to access, and can also be performed bedside inside the hospital or in the community [20].

Given the emerging role of ultrasonography, standardization of the method has recently been proposed. However, a global evidence-based consensus is still absent [21].

Ultrasonography, in the setting of screening and diagnosis of sarcopenia, has the potential to become an imaging-based tool comparable to CT, MRI to quantify body composition on the tissue level, and DXA on the chemical level.

At present, there are no pharmacological treatments available that are effective in the management of sarcopenia [22], whether they are hormone therapy or drugs. The frontier of sarcopenia treatment, especially in the elderly patient, is a personalized patient-centric approach. It is now known that there is interindividual variability at the cellular level and molecular expression [23,24,25]. This variability underlies alterations in different pathways and whose identity is the basis of the future sarcopenia management. Currently, only an adequate exercise regimen associated with correct nutritional intake (protein) has demonstrated efficacy in the management of sarcopenia. Of utmost importance is the prescription of resistance-based exercise, a diet rich in protein, and with an adequate caloric intake and vitamin supplements [26].

The aim of this paper is to update the state of the art of ultrasonographic imaging, considering the evidence in the literature, within the framework of sarcopenia.

## 2. Clinical Evaluation and Diagnosis

Sarcopenia is a common condition in the elderly, although it may also occur in young adults. Sarcopenia has multiple definitions and criteria, developed by major research groups (Table 1).

**Figure 1:**
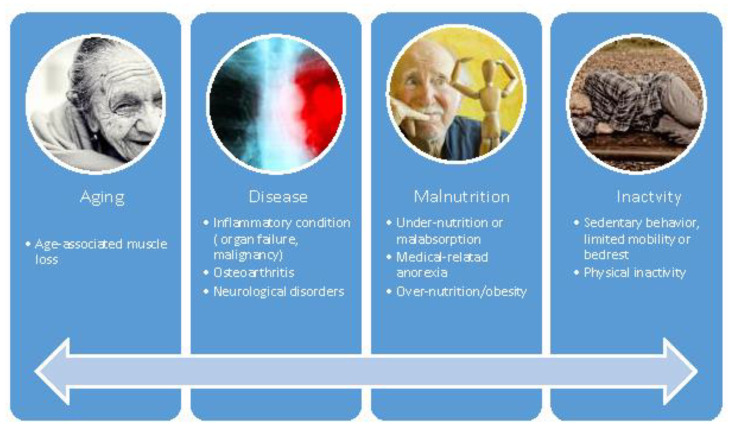
Factors that cause and worsen muscle quantity and quality are classified as primary (aging) and secondary (disease, inactivity, and poor nutrition).

The cause of sarcopenia can be identified only in some cases, in others, none may be recognized. Therefore, in clinical practice, primary sarcopenia can be discerned from secondary one concerning the underlying cause. Primary or age-related sarcopenia is when no other cause other than aging itself is detected. In many older persons, the etiology of sarcopenia is multifactorial (Figure 1), so it may not be possible to categorize it. This situation is consistent with the recognition of sarcopenia as a geriatric multifaceted syndrome [1]. In particular, some causes associated with the pathophysiological mechanisms are anorexia, inflammation, hypogonadism, lack of activity, hypovitaminosis D, loss of motor neurons, insulin resistance, poor blood flow to the muscle, mitochondrial dysfunction, and genetics [6].

The staging of sarcopenia is a concept that can help guide the clinical management of the disease. EWGSOP suggests a conceptual distinction in stages: ‘presarcopenia’, ‘sarcopenia’, and ‘severe sarcopenia’. The presarcopenia is characterized by low muscle mass with no impact on muscle strength or physical performance. This phase can only be identified by techniques that measure muscle mass accurately and regarding standard populations. The sarcopenia stage is characterized by low muscle mass in addition to low muscle strength or physical performance. Severe sarcopenia is the stage identified when all three criteria of the definition are met (low muscle mass, low muscle strength, and low physical performance) (Table 2).

Recognizing the stages of sarcopenia can aid in selecting treatments and setting appropriate recovery goals. Clinically, sarcopenia can have an acute onset or present characteristics of chronicity, for which a cut-off of 6 months has been established to distinguish acute sarcopenia (duration of fewer than 6 months), usually related to acute disease or injury, from chronic sarcopenia (duration of more than 6 months). The latter typically occurs in patients with chronic and progressive disease. It is valuable to periodically assess sarcopenia in increased-risk individuals, thus facilitating early intervention [27]. The different diagnostic approaches and cut-off values of sarcopenia have a critical impact on the epidemiology of the disease, especially worldwide. Several studies have examined the divergence in prevalence and factors associated with sarcopenia defined by current published consensus criteria and found significantly divergent results. An example concerns the prevalence of sarcopenia defined by EWGSOP2 (men: 6.5%; women: 3.3%), which appears to be lower than that defined by EWGSOP1 (men: 22.3%; women 11.7%) due to the new diagnostic criteria and more specific and selective threshold values belonging to the EWGSOP2 study [5]. Further studies will be necessary to arrive at a diagnosis of sarcopenia based on cut-offs and unambiguous criteria recognized at the international level through which concrete epidemiological data can be drawn.

Although the debate on the best modality of identifying sarcopenia is still open, diagnostic methods and tools for measuring the outcomes and characterizing sarcopenia are substantially shared by dedicated research groups.

They can be grouped into:

- Screening methods, such as the administration of a simple questionnaire. SARC-F is an example, which consists of a few questions, to investigate the crucial aspects of the interaction between the subject and the surrounding environment (muscle strength; need for assistance in walking; difficulty getting up from a chair; difficulty climbing stairs; falls in the last year). Each of these items is assigned a value from 0 to 2. The total score is a maximum of 10. SARC-F ≥4 is associated with a limitation in physical activity and predict a high risk of adverse events.
- Methods for studying muscle strength, such as the handgrip strength test and the chair to stand test. In 2018, the EWGSOP2 established new cut-offs for the diagnosis of muscle strength deficiency compatible with the diagnosis of probable sarcopenia, values below 27 kg for men and 16 kg for women at the handgrip strength test [1]. Furthermore, if the patient is unable to use the dynamometer or it is not available, it is recommended to perform the chair stand test, which measures the time necessary for the patient to get up five times. Recent studies have shown that for every 1-s increase in test performance, the probability of sarcopenia increases by 8% in older women [28]. The cut-off of 13 s showed the best balance between sensitivity and specificity, with values higher than compatible for the diagnosis of sarcopenia.
- Methods for the study of physical performance such as short physical performance battery (SPPB), gait speed and time up and go (TUG) test. SPPB and the gait speed are considered the most reliable tests. The first assesses balance, walking, strength, and muscle endurance by evaluating 3 tasks: maintaining an upright position for 10 s with the feet parallel, in semi-tandem (toe positioned laterally to the heel) and tandem (toe positioned behind the heel); the time taken to cover 4 m (gait speed); the sit to stand test. SPPB score ≤ 8, is defined by the EWGSOP as poor muscle performance. However, measuring gait speed may be sufficient to identify impaired muscle performance in clinical practice and the research field. A walking speed < 0.8 m/s is considered a sign of limitation of mobility. TUG is a simple test for the evaluation of the functional capacity of the patient. The test consists of measuring the time taken by the subject to get up from a chair, walk ten feet, turn around, return to the chair, and sit down again. A time greater than 20 s to perform the test is highly predictive of poor physical performance.
- Qualitative and quantitative methods of measuring muscle mass using different imaging techniques that analyze body composition: computed tomography (CT), and from nuclear magnetic resonance (MRI) dual-energy X-ray absorptiometry (DXA), analysis of bioelectrical impedance (BIA), and muscle ultrasound. CT and MRI ensure a precise analysis of different body tissues and are considered the gold standard for measuring muscle mass, but there is no consensus on cut-off values for sarcopenia [12,29,30,31].

CT is a technique that can be used to study muscle tissue using ionizing radiation. The qualitative and quantitative study of muscle tissue can be carried out by selecting an area of interest and then deriving muscle study parameters, such as cross-sectional area CSA. It is also possible to assess muscle and whole-body composition derived from CSA [32]. In most studies, the assessment is performed at the L3–L4 level so that psoas, paraspinous and abdominal muscles are included in the same scan.

Recently, some studies have shown that through this imaging technique it is possible to detect low muscle, both in patients with normal and high body weight, as well as predict the same prognosis if suffering from sarcopenia [33,34].

However, because of the high costs and poor availability and repeatability of the same results, as well as the poor comfort for patients, these imaging tools are not applicable in daily clinical practice and should not be prescribed to measure muscle mass, except in the context of research protocols [35]. MRI is a technique that allows the evaluation of muscle tissue composition without the use of ionizing radiation. It is a method with excellent accuracy; however, it is expensive and not always available. MRI allows a quantitative and qualitative assessment of the muscle, detecting abnormalities such as fibrous or adipose infiltration [36]. Currently, MRI in the diagnosis of sarcopenia is used only for research purposes [35].

DXA or dual-energy X-ray absorptiometry is a more affordable and accessible technique compared to MRI and CT. DXA exposes to few radiations and can estimate ASM within a few minutes. For these factors, it is the most widely used method in the clinical setting. However, DXA is still a method that can only be used in the hospital and may be affected by variations based on hydration [1,37]; moreover, DXA offers only quantitative evaluation on muscle tissue. DXA is more easily accessible but less accurate than MRI and CT [1]. This method has the advantage of collecting data on body composition by defining the amount of fat mass and lean mass in the different body areas, as well as the possibility of exposing the patient to a low dose of ionizing radiation with management costs and repeatability significantly lower than those of the CT exam. Furthermore, it is precisely based on this imaging technique that one of the first diagnostic criteria of sarcopenia was identified, represented by the skeletal muscle mass index (SMI) and defined as the ratio of appendicular muscle mass (appendicular skeletal muscle mass, ASM), measured by DXA, and the square of height in meters (ASM/h2). SMI values below −2 SD compared to those of a young adult population are considered diagnostic of sarcopenia [38]. Furthermore, DXA has the most standardized cut-offs for sarcopenia.

Bioelectrical impedance analysis (BIA) is a method of estimating muscle mass based on the electrical conductivity of the body. It represents a low-cost method, easily repeatable and usable even at the patient’s bed. BIA does not measure muscle mass directly, but it derives from it an estimate of the same, based on the electrical conductivity of the whole body, using for this purpose, a conversion equation, calibrated concerning the lean mass measured by DXA in a specific population [1]. However, it is a technique that is affected by age, ethnicity, and body hydration [39,40].

Although the latter two methods may represent a step forward for the study of sarcopenia, according to the EWGSOP [1], both BIA and DXA have a certain degree of imprecision and inaccuracy that limit their use in clinical practice. The measurements of the DXA may differ according to the manufacturer of the machine and their degree of concordance with the gold standard techniques may depend on age and sex. The accuracy of the BIA, on the other hand, strongly depends on the type of equations used by the machine for estimating the appendicular skeletal mass, which is still incorrect for the study in the elderly population and whose result depends on the state of hydration, with the fluid overload, which acts as a confounding factor.

Recent studies have shown that there is a high degree of correspondence between MRI, CT, DXA, and ultrasound for muscle mass assessment [1,21,22]. Muscle ultrasound is a research technique widely used to measure muscle quantity and quality. This imaging method is valid, reliable, and easy to use even at the patient’s bed. Although there are some limitations related to the accuracy and the result of the examination still strongly linked to the high inter-operator variability, recently, several studies have evaluated the use of this tool to support the diagnosis of sarcopenia in the elderly. The SARCUS panel, for example, proposed a protocol for using ultrasound in the assessment of sarcopenia, including measurement of muscle thickness, cross-sectional area, fascicle length, and ultrasound intensity (echogenicity) [21]. According to other studies, echogenicity correlates with the quality of muscle tissue, as the portion of tissue unable to undergo contraction appears to show increased echogenicity, a sign of myosteatosis formation [41,42]. Therefore, despite other imaging methods, ultrasound has the advantage of being able to evaluate both muscle quantity and muscle quality.

## 3. Skeletal Muscle Ultrasound in Sarcopenia

To diagnose sarcopenia in the elderly, evaluating skeletal muscle mass loss is one of the main elements. Although in recent years the importance of associating functional parameters that were to evaluate the loss of muscle performance in the elderly has been emphasized [1], the diagnosis of sarcopenia cannot ignore the precise measurement of skeletal muscle mass loss, both in quantitative and qualitative terms [13,43]. Therefore, the simultaneous evaluation of both these parameters is recommended in the muscle mass measurement phase. On the one hand, diagnostic methods, such as DXA and BIA, are limited to providing only quantitative information about the skeletal muscle structure; on the other hand, CT and MRI, while offering an overview, are burdened by the limitations in the field of everyday clinical practice. In this sense, skeletal muscle ultrasound offers a valid alternative, as it is closely correlated with MRI [44,45], with CT [46] and DXA [47,48] in terms of measuring skeletal muscle mass and at the same time offering both quantitative and qualitative information. Although further studies on the standardization of ultrasound measurement methods are needed, the first step in this direction has been provided by the SARCUS study (SARCopenia through UltraSound) [21].

Ultrasonography (US) can effectively assess the quantity and quality of muscle tissue and has a certain degree of correlation with DXA in muscle study [48,49]. Some US parameters measure muscle quantity, such as muscle volume (MV), cross-sectional area (CSA), and muscle thickness (MT). Other parameters describe the muscle qualitatively, for example, echo intensity (EI), pennation angle (PA), fascicle length (FL), and physiologic cross-sectional area (PCSA). There are some experimental approaches, such as the measurement of vascularity.

These parameters can be studied at various “regional sites”; however, the one most frequently used site is the anterior compartment of the thigh. In addition, sarcopenia is not uncommonly site-specific. Muscle loss in the lower limbs is more pronounced in the mentioned district [50,51].

The technique involves supine positioning of the subject with legs extended. A linear probe (5–12 MHz) is used, with a fixed gain, and transverse and longitudinal images are taken at the quadriceps femoris. CSA, MT, and EI are assessed on transverse images. PA and FL are measured longitudinally. MT can be performed on any muscle; firstly, the evaluation involves the quadriceps femoris; CSA is evaluated mainly in the rectus femoris. MT and CSA have a good degree of correlation on muscle quantity with the other main methods for studying muscle (MRI, CT, and DXA) [41,45,52]. However, CSA or MT are subject to variability in the muscle explored, to “regional” sarcopenia. Not all anatomical regions undergo muscle loss at the same rate, so there may be some degree of discordance.

Among qualitative measures, muscle echo intensity (EI) is a parameter that has been recently studied. Granted that the amount of muscle does not directly correlate with function [29,51], muscle echogenicity provides helpful information about the presence of inflammation, fibrosis, and adipose tissue infiltration [53]. Increased intramuscular adipose tissue, also referred to as “myostatosis”, is a finding present in both aging-related sarcopenia and cancer cachexia [54]. These tissue rearrangements can cause an increase in muscle echogenicity, similar to findings in myopathies [55].

In addition, there appears to be a correlation between echo intensity and muscle strength, gate speed, and sit-to-stand test [56,57,58].

Alterations in architecture are crucial in the genesis of force and are parameters related to muscle function.

**Figure 2:**
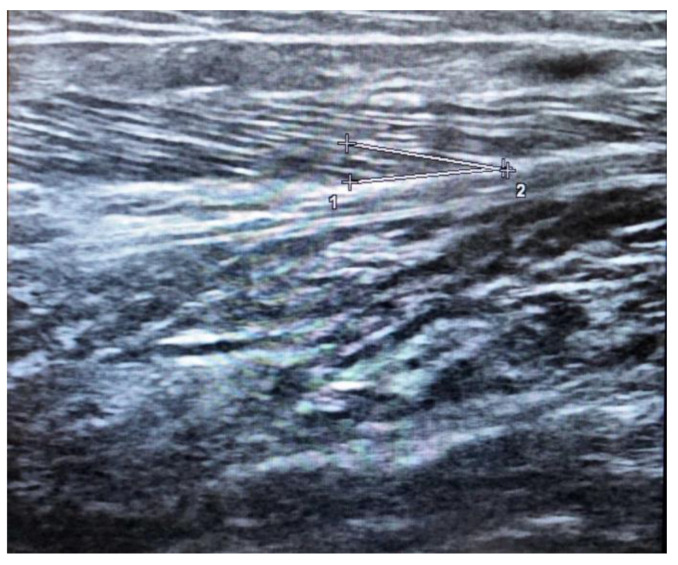
Ultrasound estimation of Pennation angle in the medial gastrocnemius given bu deep aponeurosis (1-2) and fascicle region (+).

The pennation angle is formed by the insertion of muscle fibers at the level of the deep and superficial aponeurosis in most locomotor muscles, such as the medial gastrocnemius (Figure 2). The pennation angle and fascicle length are generally evaluated at the level of the gastrocnemius medialis. These two techniques allow for information on the structure and architecture of the muscle and therefore allow for a correlation with the genesis of muscle strength [30].

However, these parameters are highly dependent on the technique used to scan. The fascicle length is the distance between the intersection composed of the superficial aponeurosis and the fascicle and the intersection composed of the deep aponeurosis and the fascicle.

These parameters present issues related to their use in long muscles. In such sections, the muscle fibers are longer than the actual field of view of classical US probes. In order to obtain a more accurate estimation, a reliable approach is to use extended field-of-view or to calculate FL from quantitative and qualitative measurements (MT and PA) [59,60].

Recently, standardization has been proposed, but a global consensus on the technique is currently missing [21]. The technique is also dependent on several US parameters (e.g., frequency, focus, gain) and muscle thickness [30]. However, it is possible, by quantitative evaluation, to eliminate this limitation [1].

Another important assessment is the physiologic cross-sectional area (PCSA); it is obtained primarily on quadriceps femoris scans and is derived from CSA, PA, and FL. It can help to assess muscle structure and its relationship to function. This parameter also allows for a more accurate estimate of the number of muscle fibers in each section.

The fascicle length and the pennation angle and their modifications are the main structural parameters that can be measured by ultrasound in skeletal muscles [61]. Narici and colleagues have shown that aging is associated with significant changes in the ultrasound structure of the medial gastrocnemius, with a reduced length of the muscle fascicle and pennation angle [62]. A decrease in physiological cross-section is related to muscle function more than the anatomical cross-sectional area measured by CT or MRI [62]. A study showed that the pennation angle and the cross-section area of the lateral head of the gastrocnemius muscle in a group of healthy older adults were smaller than the control in young subjects [63].

Additional qualitative analysis is the study of muscle vascularization; it is generally assessed at the level of the quadriceps femoris muscle. This study allows for the identification of areas at potential risk of sarcopenia, which can be visualized as alterations in the vasculature. Microvascular damage and nitric oxide deficiency are thought to underlie the pathogenesis of sarcopenia [64,65]. However, this technique requires a high level of expertise, data processing software, and special materials.

Finally, muscle stiffness is the relation between the possible degree of deformation and compression of the muscle [61]. These factors are determined by connective tissue, such as extracellular matrix collagen, which provides passive tension, and muscle contraction, which produces active tension. A recent study has suggested that changes in muscle stiffness, as measured by elastography, could be linked to muscle weakness [62].

EWGSOP2 included in the definition of sarcopenia the detection of a poor quantity and quality of muscle tissue. Thus, the combined evaluation of quantitative (CSA and MT) and qualitative (EI, FL, and PA) parameters could be important to identify sarcopenia; US evaluates in a simple and easily repeatable way any changes over time, both in terms of the evolution of sarcopenia and in terms of evaluation of the response to a given intervention. The use of additional biomarkers complementary to the US could increase the accuracy of the method itself [66].

## 4. Regional or “Site-Specific” Sarcopenia in Ultrasound

Significant loss of total muscle mass is a late event in the natural history of sarcopenia, subsequent even to the loss of physical performance. In each anatomical region, there is a different age-related rate of decline in muscle mass [67]. Indeed, sarcopenia mainly affects the muscles of the lower limb rather than those of the upper limb [68]. Therefore, there may be some muscle areas that are affected by sarcopenia and others that are not. This phenomenon is known as “regional” or “site-specific” sarcopenia. An age-related decrease in the thickness of the quadriceps femoris and abdominal muscles has been found in both Japanese and healthy Caucasian adults [69,70]. Furthermore, in the thigh muscles of older individuals, a decrease in ultrasonographically estimated cross-sectional measurements was demonstrated and confirmed by the results obtained by CT [51]. On the contrary, other anatomical sites, such as the upper limbs, would not seem to be affected by this phenomenon [69,70].

Recent studies have brought to light that a decrease in the thickness of some specific muscles, such as rectus femoris and rectus abdominis, are early changes. These, together with the assessment of muscle architecture, may contribute to the diagnosis of sarcopenia in patients with reduced physical performance, i.e., low gait speed/grip strength [71]. In case of a reduction in only one of these two performance parameters, we refer to regional sarcopenia. Recently, Sanz-Pariz et al. have observed that some muscle districts can have a correlated reduction in thickness; moreover, a significant reduction in each district was associated with a functional deficit related to the reduced muscle function. Specifically, the biceps brachialis showed decreased ability to feed itself and the quadriceps femoris showed decreased mobility [72].

## 5. Conclusions

The application of ultrasound in the assessment of sarcopenia is helpful, especially for detecting muscle failure in older patients not being influenced by the presence of acute and chronic disease and fluid balance. Despite small intra and inter-individual variability, this technique could characterize sarcopenia through the study of qualitative and quantitative parameters measured in different muscles groups, particularly in lower limb muscles. Ultrasound provides a quantitative and qualitative study of muscle, has several modalities, and can study the muscle tissue of multiple anatomical regions. For the evaluation of sarcopenia, state of the art ultrasonography had the potential to be comparable to the other main methodologies. The recent attempts to standardize the technique, the low cost, the simplicity of execution, the absence of ionizing radiation, the reproducibility, and repeatability make it a possible method to be used in the near future for the diagnostic assessment and treatment response in sarcopenia. Further studies will be needed to confirm and standardize the use of the methodology in a clinical setting.

In conclusion, having limited options for sarcopenia management, the best strategy is prevention and early diagnosis. With this perspective, determining the future role of ultrasound in this field will be crucial.

## Figures and Tables

**Table 1:** Definitions and criteria of five major research group.

International Definitions of Sarcopenia
2010 European Working Group on Sarcopenia in Older People “Sarcopenia is a syndrome characterized by progressive and generalized loss of skeletal muscle mass and strength associated with an increased risk of adverse events such as disability, poor quality of life and death […]”
2011 International Working Group on Sarcopenia “Sarcopenia is a age-related loss of muscle and function. Is a complex syndrome that is associated with isolated loss of muscle mass or associated with increased fat mass [...]”
2011 Society for Sarcopenia Cachexia and Wasting Disorders “Sarcopenia is a syndrome characterized by reduction in muscle mass associated with limitation in walking, not resulting from specific pathologic conditions or cachexia [...]”
2014 Foundation for the National Institutes of Health Sarcopenia Project “Sarcopenia is a functional limitation in the presence of reduced weakness (reduced strength) as a consequence of reduced muscle mass [...]”
2014 Asia Working Group for Sarcopenia “Sarcopenia is a recently recognized geriatric syndrome characterized by age-related decline in skeletal muscle plus low muscle strength and/or physical performance […]”

**Table 2:** Definition of sarcopenia by European Working Group on Sarcopenia in Older People (EWGSOP2).

Criteria	Test and Cut-Off	Diagnosis
- Low muscle strength by chair stand and grip strength *	Grip strength (males) < 27 kg Grip strength (females) < 16 kg Chair standing > 15 s for five rises	Probable Sarcopenia
- Low muscle quantity or quality **	ASM (males) < 20 kg ASM (females) < 15 kg ASM/height2 (males) < 7.0 kg/m^2^ ASM/height2 (females) < 5.5 kg/m^2^	Sarcopenia
- Low muscle performance ***	Gait speed ≤ 0.8 m/s Short Physical Performance Battery (SPPB) ≤ 8 points score Timed Up-and-Go Test ≥ 20 s 400 m walk test, noncompletion or ≥6 min for completion	Severe Sarcopenia

* Probable sarcopenia is identified by Criterion 1 (Low muscle strength). ** The diagnosis is confirmed by additional documentation from Criterion 2 (Low muscle quantity or quality). *** If Criteria 1, 2, and 3 (poor physical performance) are all met, sarcopenia is considered severe. ASM: appendicular skeletal muscle mass.

## Data Availability

Not applicable.
